# Linking hypertension and TyG-WC circumference index among Indian adults: A cross-sectional study

**DOI:** 10.6026/973206300214318

**Published:** 2025-12-15

**Authors:** Arijita Banerjee, Shirin Dasgupta, Arindam Ghosh

**Affiliations:** 1 Department of Physiology, Dr. B.C. Roy Multi-speciality Medical Research Centre, IIT Kharagpur, India; 2 Department of Pathology, Dr. B.C. Roy Multi-speciality Medical Research Centre, IIT Kharagpur, India; 3 Department of Biochemistry, Dr. B.C. Roy Multi-speciality Medical Research Centre, IIT Kharagpur, India

**Keywords:** Hypertension, diabetes, pre-diabetes, triglyceride indices, triglyceride-glucose indices

## Abstract

Prediction of hypertension using indicators such as triglyceride-glucose - waist circumference (TyG-WC) index among Indian adults is gaining momentum in healthcare prognosis and diagnosis for adequate treatment. Therefore, it is of interest to evaluate the TyG-WC index as a non-invasive predictor of hypertension in Indian adults aged 18-45 years. Hence, a cross-sectional study was conducted involving 58 newly diagnosed hypertensive subjects and 72 healthy controls. Independent variables included weight, BMI, WC, TyG and TyG-WC, while hypertension served as the dependent variable, with adjustments for lifestyle and biochemical covariates. Logistic regression and ROC curve analyses were used to assess the association and predictive accuracy of these biomarkers for hypertension. The TyG-WC index demonstrated the strongest predictive power for hypertension (AUC = 0.678, 95% CI: 0.662-0.696, P < 0.001), indicating a significant correlation in young and middle-aged adults.

## Background:

Among the most potent risk factors for fatal cardiac arrest is hypertension (HTN), where the primary cause of disease and mortality as a result of the secondary consequences of high arterial blood pressure is cardiovascular disease brought on by HTN [1]. As a result of affecting ventricular systole and diastolic filling, hypertension causes left atrial dilatation, left ventricular hypertrophy and impaired ventricular relaxation, or "diastolic dysfunction," which raises the risk of heart failure (HF) [2]. Chronically high blood pressure not only causes detrimental structural alterations in the myocardium but also impairs the autonomic control of blood pressure thus leading to spontaneous generation of irregular rhythm [3]. Physicians currently recommend antihypertensive medication, appropriate dietary modifications and a regular exercise regimen for patients with hypertension and heart disease caused by hypertension [4]. Only about 30 to 35% of patients are reported to benefit from these treatments, despite the fact that their goal has been to reduce the rise in arterial blood pressure. When compared to patients with non-resistant hypertension, those with resistant hypertension have a fourfold higher risk of cardiovascular events and do not respond to three or more antihypertensive medications [5]. A number of factors, such as physical activity, depression, marital status, obesity and salt intake, are linked to hypertension while one of the main contributing factors to the growing incidence of hypertension in these individuals is age [6, 7-8]. The triglyceride-glucose index (TyG index) and its several variables, such as the triglyceride glucose-waist circumference (tyg-WC), are intriguing non-invasive indicators of insulin resistance (IR), whose increased levels indicate abnormalities in metabolism [9]. The emergence of cases with a recent diagnosis of hypertension in the Chinese adult population is known to be independently associated with waist circumference (WC) [10]. Research specifically corroborating the hypertension risk indicator related to TyG indices in Indian adults is currently lacking, though [11]. Therefore, it is of interest to ascertain the TyG-WC index's efficacy as a predictor of hypertension, it is of interest to describe the connection between the index and adult hypertension between the ages of 18 and 45.

## Materials and Methods:

## Study design:

A cross-sectional study was carried out in hospital set up associated with IIT Kharagpur, among hypertensive subjects who were attending an out-patient department.

## Study participants:

According to Gupta *et al.* the prevalence of hypertension in India is 33% of the adult population. The formula (Zα/2)2 PQ / L2 was used to calculate the sample size, with a 95% CI and 5% precision [12]. In this manner, we were able to recruit 58 newly diagnosed hypertensive participants and 72 healthy age and sex matched controls after considering exclusion criteria for our study through simple random sampling in the age group of 18 to 45 years.

## Data collection:

Sociodemographic information such as age, sex, marital status, educational attainment employment status, physical activity and any history of addiction were all included in a semi-structured questionnaire that was available in Bengali and Hindi. Questions about duration of diagnosed hypertension and drugs were posed. Comorbidities such as diabetes, asthma, COPD, obesity, coronary artery disease, metabolic disorders, autoimmune disorders and any neurological conditions were investigated and excluded from the study. A minimum of two blood pressure readings (SBP≥130mmHg and/or DBP≥80mmHg) obtained by a medical professional by validated digital sphygmomanometer at least two to four weeks apart were required to diagnose hypertension. Venous blood was drawn from the subjects under aseptic precautions after an overnight fasting. Blood was drawn in fluoride-oxalate tubes for estimation of plasma glucose and in serum separator tubes for serum lipid profile and serum creatinine estimation. Samples were centrifuged for 10 minutes at 3000 rpm to separate plasma and serum. The estimations were done on the Roche Cobas C311 automated clinical chemistry analyzer. Plasma glucose was determined by hexokinase method, serum lipid profile (total cholesterol, triglycerides, HDL-C) by enzymatic colorimetric assays and serum creatinine using the Jaffe kinetic method, as per the instructions of the manufacturer.

## Study variables:

The non-invasive biomarkers in this study served as independent variables which include Weight, BMI, WC, TyG [In{(fasting triglycerides * fasting glucose)/2}] and TyG-WC (calculated as TyG *WC) [13]. The dependant variable in the study was hypertension. Sociodemographic data, lifestyle factors, serum biochemicals, anthropometric measurements, physical activity and addiction habits are among the covariates of interest chosen for this study. The metabolic equivalent of task (MET)/week was calculated by multiplying the total minutes spent on different activities each week by their corresponding metabolic equivalents in order to assess physical activity levels.

## Data analysis:

For continuous variables, the data are shown as Mean±SD and for categorical variables, as frequencies (percentages). To find differences that are statistically significant (P <.05) between groups, the chi-square test was employed. The relationship between independent variable and hypertension was investigated using both unadjusted and adjusted linear regression analysis. Potential confounding variables such as age, gender, race, BMI, employment, physical activity and smoking or alcohol use were all taken into account in the adjusted model. To assess the effectiveness of biomarkers in enhancing the detection of hypertension, Receiver Operating Characteristic (ROC) curves were created. The area under the curve (AUC), cut-off values, specificity, sensitivity and area under the curve (AUC) were noted. We used the Mann Whitney U statistics test to compare the ROC indices of different biomarkers.

## Ethical consideration:

The research has been authorised by the board of the institutional ethics committee (IIT/SRIC/SH/ISN/2024/259). All of the participants in the study sample gave their informed consent.

## Results:

Table 1 (see PDF) provides specifics about the study population's baseline characteristics. Participants were 37 years old on average and 92 of them were men, accounting for 70.76% of the total. This group had a weighted incidence of hypertension of 44.6%. Males, unemployed individuals, smokers and drinkers were more likely to have hypertension. Additionally, a higher prevalence of hypertension was seen in those with higher measurements across a variety of indices, including the BMI, WC and TyG indices. Additionally, the group with hypertension has higher rates of low physical activity. The levels of biochemical markers such as creatinine, triglycerides and glucose were also higher in hypertensive individuals. BMI-Body mass index, WC-waist circumference, TyG- Triglyceride glucose index, TyG-WC- Triglyceride glucose waist circumference index, SD-standard deviation, MET- metabolic equivalents, SBP-systolic blood pressure, DBP- diastolic blood pressure. The association between the prevalence of hypertension and biomarkers was evaluated using weighted logistic regression multivariate analysis; both 95% CI and odds ratios (OR) were used to present the results. Two models were created to take into consideration potential covariates: the first model represented as 1, was unadjusted model while the second one was modified to take into account factors like age, gender, education, drinking, smoking, sedentary behaviour and serum biochemical parameters. To investigate their association with hypertension, biomarkers were employed as both continuous and categorical variables in a weighted multivariate logistical regression model (Table 2 - see PDF). BMI (Model 1: OR=1.10 (0.92-1.21), WC (Model 1: OR=1.12 (0.96-1.21), Plasma glucose (Model 1: OR= 1.10 (0.86-1.22) and Triglycerides (Model 1: OR = 1.16 (1.12-1.22) were significantly linked to the risk of hypertension but displayed an unsteady correlation in the adjusted model. TyG (Model 1: OR=1.16 (1.12-1.22)) and TyG-WC (Model 1: OR=1.27 (1.18-1.34)) are all significantly linked to the risk of hypertension when examined as a continuous variable. They all displayed a steady correlation that persisted in the modified model (p<0.001). Additionally, the hypertension risk increased with higher biomarker quartiles in the group comparison, with the lowest quartile serving as the reference (all P for trend<0.001). The value of the four biomarkers was evaluated using ROC analysis in order to improve the detection of common hypertension (Table 3 - see PDF). The most potent accuracy for prediction was shown by TyG-WC, with an AUC of 0.678 (95%CI: 0.662-0.696) for the detection of prevalent hypertension, which was much greater than that of other biomarkers (P<0.001).

## Discussion:

Currently affecting one-third of the population, hypertension is the primary reason for early mortality globally and dramatically increases the cardiovascular as well as cerebrovascular risk. Therefore, the prevention of hypertension depends on early prediction and prompt intervention. Treating primary hypertension becomes more difficult in elderly patients due to decline in autonomic function and arterial elasticity with age. A decrease in the bulk of muscle and the build-up and the distribution of visceral adipose tissue in older people frequently complicate the link between obesity and hypertension. One of the primary causes of adult hypertension is metabolic issues like obesity and hyperlipidaemia, as adolescents and young adults are less inclined to have other chronic diseases. As a result, biomarkers that are relevant for youth might not be for the elderly [14-15]. Higher prevalence of hypertension was observed in those with higher measurements across a variety of indices, including the BMI, WC and TyG indices. Additionally, the group with hypertension has higher rates of low physical activity, which is in accordance with previous research [16, 17]. Currently, a wide variety of biomarkers are employed to predict hypertension early on. A growing body of research indicates that conventional biomarkers, like waist circumference and BMI, can assist public health officials in creating plans to mitigate incidence of hypertension in the individuals. These conventional serum markers have shown themselves to be useful instruments for hypertension screening in outpatient settings. However, traditional obesity biomarkers are unable to accurately reflect the body's metabolic status because they are unable to distinguish between lean and fat body mass. As a result, they are unable to precisely reflect the risk of hypertension. The "obesity paradox," as it is commonly known in clinical practice, states that people with increased BMI or waist circumference do not have an elevated risk of hypertension and cardiovascular disease. Gender, age, race, or illness types have no bearing on this universal paradox. Since multivariate analyses show virtually no correlation between BMI, weight, WC and hypertension, our research has validated this phenomenon. These biomarkers have had limited clinical use as a result of this contradiction. In order to predict the risk of hypertension in a clinical setting, we must thus find more reliable biomarkers [18, 19, 20-21]. We found that there is a particularly strong correlation between adult hypertension and TyG/TyG-WC. In line with the findings of a few earlier studies, our analysis of the indices of TyG and TyG-WC showed that TyG-WC had the powerful correlation with the likelihood of hypertension [22, 23]. Triglyceride and glucose levels are included in the TyG index, which enables it to concurrently represent the state of lipolysis. As a result, TyG is linked to the death rates and acts as a marker for atherosclerosis and CVD. By raising adipokine secretion and sympathetic nervous system activity, insulin resistance may have a synergistic effect on the obesity-hypertension association. This illustrates the basic process by which TyG can represent the population's risk of hypertension. It may be possible to detect hypertension more accurately with the TyG-WC index as shown in previous research study [24]. It provides a more accurate representation of the body's metabolic state by combining TyG and WC. Increased renin angiotensinogen aldosterone system (RAAS) activation, arterial stiffness and insulin resistance are all associated with elevated TyG levels and are linked to hypertension. This combined method offers a thorough understanding of metabolic status and is strongly linked to hypertension, which helps create efficient management plans for the condition [25].

## Strength and limitations of the study:

Multidimensional information such as demography, physical characteristics, plasma and serum markers, addiction history and physical activity are among the covariates we included. Many variables that could influence the risk of hypertension were taken into account and the results held up well after controlling for a number of variables, demonstrating the validity of the conclusion. Finally, the TyG-WC parameter is useful for clinical application because it is simple to obtain and compute. In a cross-sectional study, we can only vouch for the association between two variables. Additional research is required to elucidate the causal relationship between hypertension and TyG-WC. Although we made an effort to include a variety of biomarkers, TyG-WC demonstrated the likelihood of developing hypertension risk more significantly; however, its area under curve value was only about 0.7, suggesting that its predictive performance was moderate. Better biomarkers of prediction for clinical use must be further investigated in the future.

## Conclusion:

Adults between the ages of 18 and 45 had a significant correlation between TyG-WC and hypertension. Hence, public health agencies may use this information to enhance blood pressure control plans.

## Author contributions:

AB- Data collection, manuscript writing. SD, AG, AB- Data analysis. All authors contributed to manuscript revision, read and approved the submitted version.

## Funding:

The authors declare that the study was conducted in the absence of any financial support.

## Data availability and sharing policy:

The data supporting this study's findings are available from the corresponding author upon reasonable request.
